# Conservation and Remodeling of Alternative Splicing Landscapes in the Evolution of *Panax*

**DOI:** 10.3390/plants14213301

**Published:** 2025-10-29

**Authors:** Jing Zhao, Xiangru Meng, Peng Di, Junbo Rong, Hongwei Xun, Siwen Zheng, Juzuo Li, Jian Zhang, Ying-Ping Wang

**Affiliations:** 1 College of Chinese Medicinal Materials, Jilin Agricultural University, Changchun 130118, China; zhaoj878@nenu.edu.cn (J.Z.); mengxiangru0814@163.com (X.M.); di@jlau.edu.cn (P.D.); 15844490775@163.com (J.R.); siwen0717@126.com (S.Z.); 2 State Local Joint Engineering Research Centre of Ginseng Breeding and Application, Jilin Agricultural University, Changchun 130118, China; 3 Key Laboratory of Molecular Epigenetics of the Ministry of Education (MOE), Northeast Normal University, Changchun 130024, China; xunhw334@nenu.edu.cn; 4 Department of Biology, School of Life Sciences, Southern University of Science and Technology, Shenzhen 518055, China; 5 Faculty of Agronomy, Jilin Agricultural University, Changchun 130118, China; 6 Department of Biology, University of British Columbia, Okanagan, Kelowna, BC V1V 1V7, Canada

**Keywords:** *Panax*, alternative splicing, polyploidization, post-transcriptional regulation

## Abstract

Alternative splicing (AS) is a widely recognized post-transcriptional regulatory mechanism that plays a crucial role in plant evolution and environmental adaptation. In this study, five representative *Panax* species were systematically analyzed to examine the evolutionary conservation and functional characteristics of AS events. Results revealed an expansion in the number of AS events and associated genes across the *Panax* species, accompanied by a genome-wide shift in splicing types from a dominance of intron retention (IR) to an increase in exon skipping (ES), alternative donor (A5), and alternative acceptor (A3) events. Splicing preferences were also found to diverge among allotetraploid species, which exhibited more complex AS patterns. The genomic features of IR and ES events, such as GC content and length of the sequence involved in AS, were highly conserved among *Panax* species of different ploidy levels (diploid vs. allotetraploid). Genes harboring conserved IR events across all five species were identified, and functional annotation indicated that these genes are primarily involved in chromatin modification and RNA splicing-related processes. This study elucidates the dynamic remodeling of AS during the evolution of *Panax* and provides important insights into the evolutionary adaptive mechanisms of AS in plants.

## 1. Introduction

Alternative splicing (AS) is a crucial post-transcriptional regulatory mechanism that significantly enhances transcriptomic and proteomic complexity and diversity by generating multiple distinct mRNA isoforms from a single pre-mRNA molecule [1,2,3]. In plants, AS plays an equally indispensable role [4]. The occurrence of different AS types, primarily intron retention (IR), exon skipping (ES), alternative donor (A5), and alternative acceptor (A3) events, varies among plant species [5,6,7]. Although IR is usually the most frequent type, AS collectively plays an equally critical role in plants [4]. This contrasts with the animals, where ES is predominant [8]. AS has been demonstrated to be integral to plant growth, development, response to various biotic and abiotic stresses (e.g., heat, drought, salinity), and the shaping of phenotypic plasticity [1,9,10]. A deep understanding of AS patterns is therefore essential for a comprehensive interpretation of gene function and its regulatory mechanisms in complex biological processes.

During evolutionary processes, AS has not only served as a key mechanism for gene expression regulation but has itself evolved, providing organisms with rich potential for environmental adaptation and functional innovation, thereby driving speciation and diversity [10,11,12,13]. Previous studies indicate that AS patterns can evolve independently of gene expression levels, offering different pathways for natural selection to act upon [14,15,16]. Whole-genome duplication (WGD), particularly allopolyploidization, is a widespread evolutionary phenomenon in plants that shapes genome structure and function [17,18,19]. By merging divergent parental genomes into a single nucleus, allopolyploidization not only increases gene copy numbers but can also lead to functional divergence of gene duplicates (sub- or neofunctionalization), the acquisition of novel functions, and rapid alterations in AS patterns [20,21,22]. For instance, extensive changes in AS patterns have been observed following WGD in species such as *Glycine max* and *Brassica rapa*, underscoring the important role of AS in the divergence of gene copies [23,24]. Analyses of AS patterns in genes encoding splicing factors, such as the SR protein family, have further revealed both conservation and flexibility across species, hinting at deeper regulatory mechanisms governed by AS [25,26].

The genus *Panax* encompasses species of significant medicinal and economic importance, including *Panax ginseng*, *Panax quinquefolius*, and *Panax notoginseng* [27,28,29]. This genus shares the core-eudicot γ hexaploidization event and has undergone additional *Panax* specific WGDs, making it an ideal model system for investigating how allopolyploidization reshapes post-transcriptional regulatory networks, particularly the evolution of AS landscapes [30,31]. However, despite the availability of genomic and transcriptomic resources for *Panax* species, the question of how allopolyploidy events have systematically influenced the breadth, complexity, and functional conservation of the AS landscape within this genus remains largely unexplored.

This study employs a comparative transcriptomic analysis of five representative *Panax* species (including both diploids and allopolyploids) and an outgroup to systematically investigate the evolutionary dynamics of AS during the evolution of the genus. By comparing AS patterns among different polyploid species, convergent or divergent evolutionary trajectories in splicing regulation are assessed to understand the impact of AS patterns. Furthermore, despite the overall dynamic nature of AS evolution, detailed analyses of AS events have revealed that some are highly conserved in molecular features and biological function and have been consistently maintained throughout the evolutionary history of *Panax*. The study aims to provide novel insights into how alternative splicing contributes to enhanced gene regulatory complexity and adaptive evolution within the *Panax* genus.

## 2. Results

### 2.1. The Expansion and Diversification of Alternative Splicing in Panax Species

**Figure 1:**
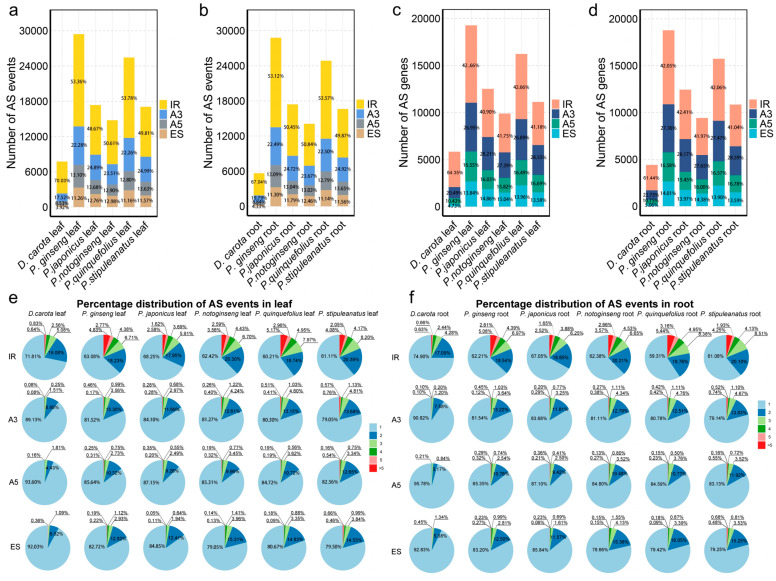
Comparative analysis of alternative splicing (AS) in five *Panax* species (*Panax ginseng*, *Panax japonicus*, *Panax notoginseng*, *Panax quinquefolius*, *Panax stipuleanatus*) and the outgroup *Daucus carota*. (**a**,**b**) Numbers and frequencies of four types of AS events (IR, A3, A5, ES) in leaf and root tissues, respectively. (**c**,**d**) Number and percentage of genes undergoing AS in leaf and root tissues, respectively, the different colors represent different AS event types. (**e**,**f**) Frequency distribution of the number of AS events per gene in leaf and root tissues, respectively. Colors indicate the number of AS events per gene: light blue (1), dark blue (2), green (3), light green (4), orange (5), and red (>5).

To investigate the role of AS in the evolution of the *Panax* genus, we utilized public transcriptome data to identify AS events in leaf and root tissues for five representative species (*P. ginseng*, *P. quinquefolius*, *Panax japonicus*, *P. notoginseng*, *Panax stipuleanatus*) and the outgroup *D. carota*. The analysis covered four AS types: IR, A3, A5, and ES. The results demonstrate a significant increase in both the number of AS events and the number of genes undergoing AS in *Panax* species compared to the outgroup *Daucus carota* (Figure 1). In leaf tissue, the five *Panax* species exhibited substantially more AS events than *D. carota* (7751 events), with *P. ginseng* showing the highest count (29,425 events), a 3.8-times increase relative to *D. carota* (Figure 1a, Table S1). A similar expansion of AS events was observed in root tissues, indicating that this increase is a systemic evolutionary feature of the *Panax* lineage rather than a tissue-specific adaptation (Figure 1b, Table S2). Furthermore, the compositional patterns of AS events have shifted. Although IR was the most prevalent AS type in all analyzed species, its proportion differed between *Panax* and the outgroup. In *D. carota*, IR events accounted for 70.3% and 67.04% of total AS events in leaf and root tissues, respectively, whereas in the five *Panax* species, IR comprised approximately 50% of AS events in both tissues (Figure 1a,b, Tables S1 and S2). Correspondingly, the other three AS types (A3, A5, ES) made higher relative contributions in the *Panax* species (Figure 1a,b, Tables S1 and S2). The number of genes undergoing AS, consistent with the expansion of AS events, was higher in the five *Panax* species than in *D. carota* (Figure 1c,d, Tables S3 and S4). In leaf tissue, 19,274 genes underwent AS in *P. ginseng*, compared to only 5838 in *D. carota* (Table S3). A similar trend was observed in roots (Figure 1d, Table S4). Additionally, IR was the dominant splicing mode across all species, followed by A3 and A5, with ES being the least frequent (Figure 1c,d, Tables S3 and S4). In summary, the AS landscape of *Panax* has been reshaped during its evolution, as reflected not only by the increased number of AS events and AS genes, but also in the greater complexity of splicing patterns, shifting from an IR-dominated mode to a more diversified profile with higher proportions of A3, A5, and ES types.

To further explore AS complexity, we analyzed the distribution of AS events per alternatively spliced gene (ASG). The analysis revealed that in all species and tissues, the vast majority of ASGs undergo only a single AS event, while the number of genes with multiple AS events (≥2) dramatically decrease as the event count increases. The decrease was most pronounced for A3, A5, and ES, whereas IR showed a large number of genes undergoing multiple AS events (Figure 1e,f, Tables S5 and S6). Although a single AS event per gene predominates across *Panax* species, AS complexity exhibits significant interspecific divergence. *P. japonicus* shows the simplest profile, with the highest proportion of ASGs with a single IR event in both root and leaf tissues; in its leaves, single IR events account for 68.25% of ASGs (Figure 1e,f). In contrast, *P. quinquefolius* is enriched for genes with multiple IR events; *P. stipuleanatus* for multiple A3/A5 events; and *P. notoginseng* exhibits a notably higher complexity in ES events (Figure 1e,f). In root tissue, only 133 ASGs with more than five IR events were observed in *P. japonicus*, whereas *P. ginseng* and *P. quinquefolius* had 397 and 360, respectively (Table S6), indicating divergence in the capacity to generate transcript diversity. Together, these species-specific patterns highlight the dynamic evolution of AS and likely contribute to differences in ecological adaptation and agronomic traits across *Panax* species.

### 2.2. Highly Conserved Molecular Features of Alternative Splicing Events Across Panax Species

**Figure 2:**
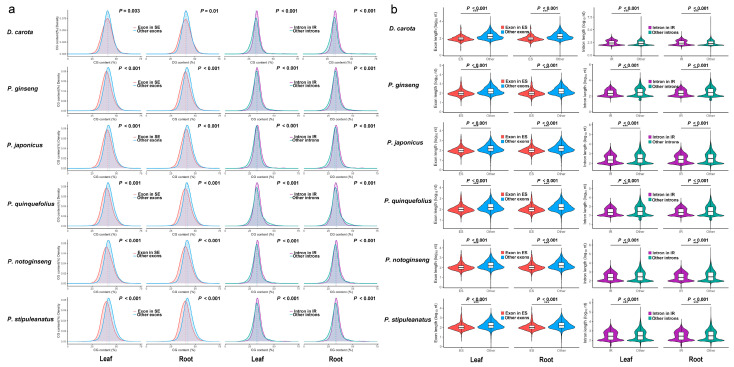
Conserved structural characteristics of sequences associated with alternative splicing in *Panax* species and the outgroup *D. carota.* (**a**) Density distribution of GC content for exons and introns in leaf and root tissues: the red curve represents exons undergoing ES events, the blue curve represents genomic background exons; the purple curve represents introns undergoing IR events, and the green curve represents genomic background introns. (**b**) Violin plots of exon and intron length distributions for ES and IR events in leaf and root tissues: red indicates exons involved in ES events, blue indicates constitutive exons; purple indicates introns involved in IR events, green indicates spliced introns. *** denotes statistically significant differences by Wilcoxon test, *p* < 0.001.

AS not only to vary across species and tissues, but also correlate with intrinsic gene and exon/intron sequence features that can modulate splicing efficiency and splice site recognition. To assess whether these sequence attributes are preserved across *Panax* species, we analyzed two AS types (ES and IR) to quantify GC content and length of the alternatively spliced elements relative to the genomic background. Notably, in both leaf and root tissues across all five *Panax* species and the outgroup *D. carota*, sequences involved in ES and IR events exhibited significant biases in GC content compared to corresponding controls. Skipped exons consistently exhibited lower average GC content than constitutive exons (Wilcoxon test, *p* < 0.01, Figure 2a), whereas retained introns displayed significantly higher GC content than spliced introns (Wilcoxon test, *p* < 0.001, Figure 2a). Additionally, the lengths of exons and introns involved in ES or IR events were significantly shorter than their constitutive counterparts (Wilcoxon test, *p* < 0.001, Figure 2b) consistent with previous study showing that short introns are more easily retained [32].

In summary, these results demonstrate that the structural characteristics of ES and IR events, specifically GC content bias and reduced sequence length, are conserved across *Panax* species. The consistent patterns observed in both diploid species (*P. notoginseng* and *P. stipuleanatus*) and allotetraploid species (*P. ginseng*, *P. quinquefolius*, and *P. japonicus*) suggest that sequence traits associated with splicing were likely established in the ancestral *Panax* lineage and have been maintained throughout subsequent speciation and polyploidization.

### 2.3. Asymmetrical Evolutionary Dynamics of Alternative Splicing in Panax

**Figure 3:**
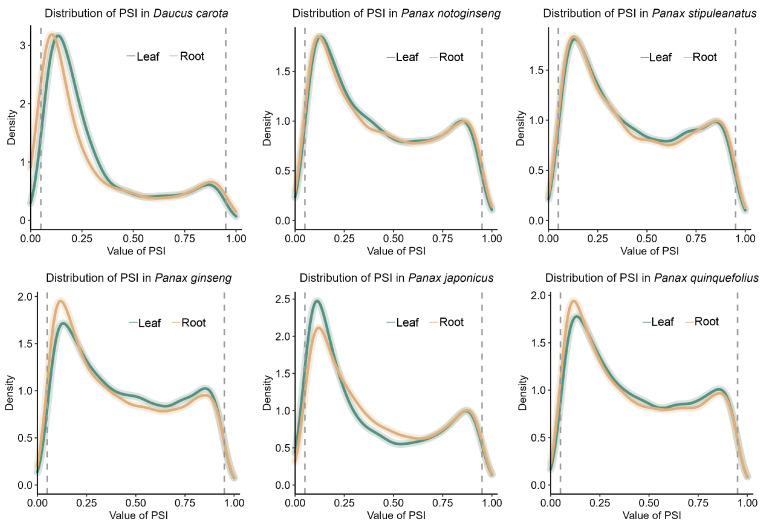
Density distributions of PSI values of AS events in leaf and root tissues of five *Panax* species (*P. notoginseng*, *P. stipuleanatus*, *P. ginseng*, *P. japonicus*, *P. quinquefolius*) and the outgroup *D. carota*. The green curve represents the PSI distribution of leaf, and the orange curve represents the PSI distribution of root.

To characterize the alternative splicing landscape across *Panax* species, percent–spliced–in (PSI) values were used to quantify alternative splicing events in all tissue samples. A systematic analysis of PSI value distribution revealed a conserved overall distribution between the leaf and root tissues within each of the five species (Figure 3). Significant differences in overall splicing patterns were observed between the allopolyploid *Panax* species (*P. ginseng*, *P. quinquefolius*, *P. japonicus*) and their diploid relatives (*P. notoginseng*, *P. stipuleanatus*), with the exception of the comparison between *P. quinquefolius* and *P. stipuleanatus* in leaf tissue, where no significant difference in PSI distribution was detected (Kolmogorov–Smirnov test [KS test], *p* = 0.106; Table 1). All other comparisons revealed statistically significant differences (KS test, *p* < 0.05; Table 1). Among the diploid species, no significant differences were detected between leaf and root tissues in either *P. notoginseng* or *P. stipuleanatus* (KS test, *p* > 0.05; Table 1), suggesting evolutionary conservation of splicing regulation within this lineage (Figure 3, Table 1). In contrast, significant differences in PSI distributions between leaf and root tissues were identified among the allotetraploid species, with the exception of the comparison between *P. ginseng* and *P. quinquefolius* in root tissue, where no significant difference in PSI distribution was detected (KS test, *p* = 0.578; Table 1). For instance, highly significant distinctions were observed between *P. japonicus* and *p. ginseng*, as well as between *P. japonicus* and *P. quinquefolius* (KS test, *p* < 0.001; Table 1). These findings indicate that despite a relatively conserved overall distribution of PSI, splicing patterns have undergone significant lineage-specific diversification following polyploidization.

**Figure 4:**
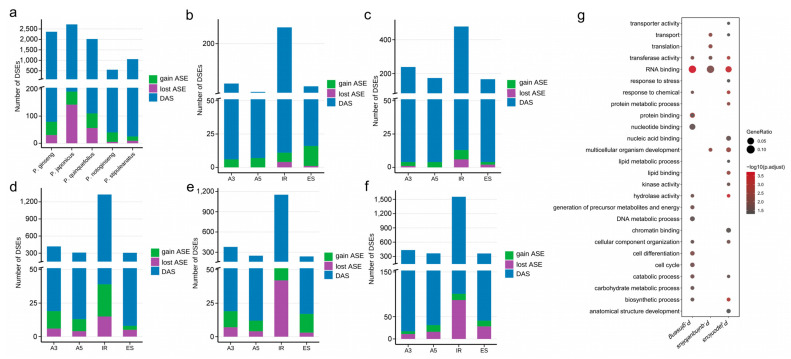
Quantitative–qualitative composition of differentially spliced events (DSEs) across *Panax*. (**a**) Total numbers of DSEs for root vs. leaf comparisons in each species. (**b**) *P. notoginseng*, (**c**) *P. stipuleanatus*, (**d**) *P. ginseng*, (**e**) *P. quinquefolius*, and (**f**) *P. japonicus*. Blue represents DAS, green represents gain ASE, and purple represents lost ASE. (**g**) Enrichment analysis of ASG in three allotetraploid *Panax* species (*P. ginseng*, *P. quinquefolius*, and *P. japonicus*). Circle size represents GeneRatio (N/M), the number of genes from our gene list found in a specific pathway (N) to the total number of genes annotated to that same pathway in the genomic background (M); color depth indicates statistical significance (−log10(*p*-adjust)).

To investigate the role of alternative splicing events in the evolution of *Panax*, a systematic comparison of differential alternative splicing (DAS) was conducted across five *Panax* species. The analysis revealed that IR events were identified as the predominant form of DAS in six species (Figure 4a–f, Tables S7–S11). Examination of gains and losses of AS events uncovered species-specific evolutionary dynamics. Although IR was the dominant event type, the ratio of its gained to lost events varied significantly among species. In *P. japonicus*, for instance, lost IR events constituted 5.60% (87/1554, Figure 4f, Table S8) of its total IR events, whereas gained events accounted for only 0.90% (14/1554, Figure 4f, Table S8). Other species exhibited a smaller disparity between the numbers of gained and lost IR events. Patterns of gain and loss for A3, A5, and ES events similarly vary by species. Notably, ES events exhibit a marked gain bias in *P. quinquefolius* and *P. notoginseng*. In *P. quinquefolius*, gained and lost ES events accounted for 5.88% and 1.26% of differential ES events (14/238 and 3/238, Figure 4e, Table S9); in *P. notoginseng*, the corresponding values were 13.89% and 0.93% (15/108 and 1/108, Figure 4b, Table S10). This pronounced gain bias suggests that the emergence of novel ES events may be a key molecular mechanism driving functional differentiation between tissues in these two species. Meanwhile, a gain bias for A5 events was also observed in *P. notoginseng* and *P. stipuleanatus*. In these species, newly gained events accounted for 7.29% (7/96) and 2.30% (4/174) of their total A5 events, respectively, with no corresponding losses detected (Figure 4b,c, Tables S10 and S11). This biased acquisition indicates that the emergence of new splice sites is an important route for increasing transcriptomic complexity and regulatory potential. In summary, these results confirm that IR is the principal source of AS diversity in *Panax* and demonstrate that the gains and losses of different splicing types have followed asymmetric, species-specific patterns.

Gene Ontology (GO) enrichment analysis was conducted for genes undergoing differential alternative splicing across three allotetraploid *Panax* species (*P. ginseng*, *P. quinquefolius*, and *P. japonicus*). Significant enrichment was observed in RNA-binding, transferase activity, transporter activity, and stress response (Figure 4g). Species-specific patterns emerged: *P. ginseng* was enriched for DNA metabolism, chromatin binding, and cell-cycle and cell-differentiation–related terms; *P. quinquefolius* for transferase activity, translation, and transport; and *P. japonicus* for stress/chemical responses, transporter activity, and kinase activity (Figure 4g). These findings reflect long-term adaptive evolution within the genus. Overall, alternative splicing events were found to be non-randomly distributed, and their functional enrichment highlights a contributory role in transcriptional regulation and species adaptation.

### 2.4. Landscape of Conserved Alternative Splicing Events in Five Panax Species

**Figure 5:**
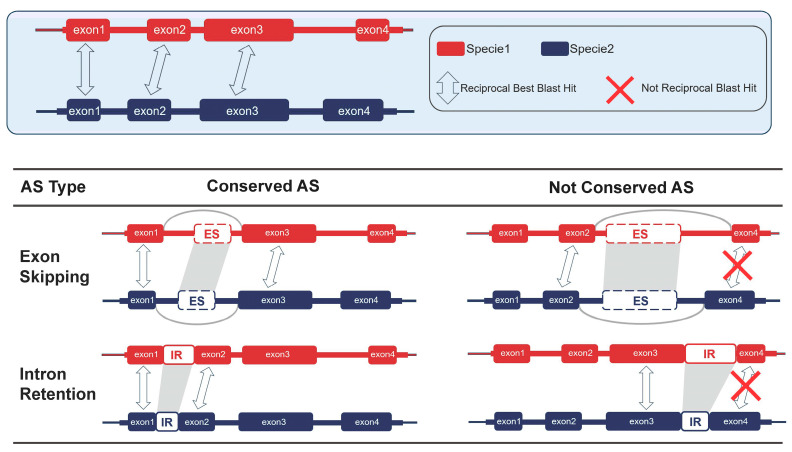
Schematic illustrating the identification of homologous exon relationships between orthologous gene pairs in *Panax* and the assessment of conservation for ES and IR. Orthologous exon pairs between Species 1 (red) and Species 2 (blue) were identified using reciprocal best BLAST hits (RBH), with homologous exons linked by gray double-headed arrows. Exons are shown as colored blocks and splice junctions as gray arcs; exons lacking RBH support are marked with a red cross. For ES, an event was considered conserved when an exon1–exon3 splice junction was detected in both species, and non-conserved when that junction was present in only one species. For IR, an event was considered conserved when intron retention (exon1–intron–exon2) was supported in both species and the flanking exons displayed clear synteny. Non-conserved IR includes cases where IR was detected in only one species, either alongside a spliced exon1–exon2 junction or with no support in the other species, or where IR was detected in both species but the flanking exons lacked sufficient reciprocal homology (non-RBH).

To investigate the evolutionary conservation of AS events within the genus *Panax*, conserved AS events were systematically identified and compared across five *Panax* species in both leaf and root tissues. Due to difference in the function and structure between IR and ES, we employ a strategy that leverages syntenic exons flanking the alternatively spliced loci to evaluate both IR and ES events (Figure 5).

In leaf tissue, a total of 480 conserved AS clusters were detected, corresponding to 2215 AS events in 1661 genes. In roots, 451 conserved clusters containing 2107 events in 1484 genes were identified (Tables S12 and S13). It was observed that a subset of genes exhibited two or more conserved AS events, suggesting that these genes may be implicated in potential core regulatory nodes. In both tissues, IR was the dominant type among conserved AS events, accounting for 90.4% (434 of 480) of the conserved clusters in leaves and 90.9% (410 of 451) in roots. In contrast, ES was considerably less frequent, representing only 9.6% and 9.1% of the conserved clusters in leaves and roots, respectively. The number of conserved cluster was negatively correlated with the number of species sharing the event. Taking the most abundant IR events in leaves as an example, 176 clusters were shared between any two species. This number decreased to 144 clusters across three species, 77 across four species, and only 37 clusters that were conserved in all five *Panax* species (Table S12). These 37 core IR clusters involved 241 genes. An almost identical trend was observed in roots, where the number of conserved IR clusters decreased from 175 (between two species) to 38 (shared by all five species), encompassing 243 genes (Table S13). ES events exhibited very limited conservation, with no clusters conserved across five species in either tissue (Tables S12 and S13).

To further examine the conservation of AS between subgenomes, the phylogenetic tree was constructed using protein sequences from *P. ginseng*, *P. japonicus*, *P. quinquefolius*, *P. stipuleanatus*, *P. notoginseng*, and the outgroup species *D. carota* (Figure S1). Within the *Panax* genus, the two diploid species, *P. notoginseng* and *P. stipuleanatus,* are positioned basally relative to the allotetraploid clade. In the three allotetraploid species (*P. ginseng*, *P. quinquefolius*, and *P. japonicus*), the homeologous gene copies are resolved into two distinct subclades, subgenomes A and B, which are sister to *P. stipuleanatus* and *P. notoginseng*, respectively (Figure S1). Further examination of conserved IR clusters shared among three or four species in leaf tissue showed that the highest abundance of conserved three-cluster events occurred in the three allotetraploid species, *P. ginseng*, *P. quinquefolius*, and *P. japonicus*, while the greatest number of conserved four-cluster events was identified in *P. ginseng*, *P. quinquefolius*, *P. japonicus*, and *P. notoginseng* (Figure S2, Table S12). These distribution patterns align with the phylogenetic relationships among these species (Figures S1 and S2). A comparable trend was detected in root tissue (Figures S1 and S3, Table S13), implying that conserved AS events in these orthologous genes may have played a role in the divergence within the genus *Panax*. To explore subgenome-level conservation, AS events in orthologous genes were analyzed across the subgenomes of the three allotetraploid species: *P. ginseng*, *P. quinquefolius*, and *P. japonicus*. Among shared conserved two-cluster events, the greatest number was observed between subgenome B of *P. ginseng* and subgenome B of *P. quinquefolius*, followed by subgenome A of *P. ginseng* and subgenome A of *P. quinquefolius*. Moreover, the counts of conserved two-cluster events between subgenome A of *P. ginseng* and subgenome A of *P. japonicus*, as well as between subgenome B of *P. japonicus* and subgenome B of *P. quinquefolius*, were consistent with established phylogenetic relationships (Figures S1 and S4). A parallel distribution was noted for conserved three-cluster events across the subgenomes of these three allotetraploid species (Figures S1 and S5). These findings further indicate that AS events may have contributed to subgenome differentiation.

To further investigate the potential functions of conserved AS events during the evolution of five *Panax* species, functional analysis was conducted on genes associated with conserved IR events. At the transcriptional and epigenetic levels, multiple chromatin-modifying enzymes were identified, including histone deacetylases (e.g., HDA6, HDA9, HDAC19) and Jumonji C (JmjC) domain-containing histone demethylases (e.g., JMJ20, JMJ21, JMJ22, JMJ30), as well as methyl-CpG-binding domain (MBD) proteins and transcription factors from the B3 and bHLH families. In post-transcriptional regulation, several core factors involved in RNA splicing and processing were detected, such as serine/arginine-rich (SR) splicing factors (e.g., *SCL30*, *SCL30A*, *SCL33*) and the key splicing regulator *SUA*. Regarding proteostasis regulation, core components of the ubiquitin–proteasome system (UPS) were revealed, including deubiquitinating enzymes (e.g., UBP5, UBP8, UBP9) and F-box proteins, along with regulatory subunits of the mitochondrial Clp protease (e.g., CLPX1, CLPX2, CLPX3). Furthermore, this set of genes was also enriched in key players involved in signal transduction (e.g., sphingosine kinases SPHK1, SPHK2), cell wall modification (e.g., prolyl 4-hydroxylases P4H1, P4H3, P4H8, P4H10), and programmed cell death (e.g., MACPF domain-containing proteins CAD1, NSL1) (Tables S14 and S15). In summary, evolutionarily conserved alternative splicing events in *Panax* species are not randomly distributed but are concentrated at critical functional nodes within regulatory networks. These findings suggest that AS may contribute to enhanced post-transcriptional regulatory capacity in polyploid *Panax* species through fine-tuning of these key genes, thereby supporting their complex regulatory networks and environmental adaptability.

## 3. Discussion

Alternative splicing (AS) is a key post-transcriptional regulatory mechanism that enhances the diversity of eukaryotic transcriptomes and proteomes, thereby providing a rich substrate for environmental adaptation and phenotypic evolution [1,33]. In polyploid plants, WGD events induce genomic shock and result in gene dosage redundancy, which is thought to require extensive transcriptomic and epigenetic remodeling to establish a new regulatory homeostasis [17,19]. However, compared to gene expression regulation, the global dynamics and functional significance of AS in polyploid evolution remain underexplored [34,35]. Phylogenetic reconstruction supported the allopolyploid origin of the allotetraploid *Panax* species, indicating their genomes were formed by the fusion of two distinct ancestral genomes [31,36]. Then, by systematically comparing diploid and allopolyploid species within the genus *Panax*, this study revealed an expansion of AS complexity following polyploidization in *Panax*, providing additional insight into the role of post-transcriptional regulation in plant adaptive evolution. Furthermore, a higher number of AS events and ASGs in allotetraploid species (*P. ginseng*, *P. quinquefolius*, and *P. japonicus*) also suggests that polyploidization may have provided more opportunities for post-transcriptional regulation. To place our findings in a broader context, we compare them with work in other polyploid crops. In *Glycine max*, a comprehensive species-level survey mapped the AS landscape on a paleopolyploid background, and later studies emphasized stress-specific remodeling rather than genus-wide conservation [23,37]. In *Brassica rapa*, genome-scale profiling and analyses of a synthetic hexaploidy versus its parents documented extensive AS and polyploidy-linked divergence, but not in a genus-wide comparison with matched ploidy [38,39]. In *Triticum aestivum,* domestication/polyploidization and abiotic stresses also reshape AS, largely at the species level [40,41]. Building on these species-focused studies, our analysis explicitly accounts for ploidy across diploid and allotetraploid *Panax* and shows that conserved AS events track both species and subgenome phylogeny at the genus scale—alongside an expansion of AS/ASGs, a shift from IR-dominated to more balanced A3, A5, ES, and divergence in tissue-resolved DAS among allotetraploids—clarifying how splicing programs diversify after polyploidization.

Changes in the composition of the AS landscape was observed among species [42,43]. While IR remains the most common type, its relative frequency decreased in *Panax* compared to the outgroup *D. carota*, and the proportions of the other three types (A3, A5, and ES) increased. This shift from an IR-dominated to a more balanced pattern may contribute to the generation of new functional isoforms, thereby potentially promoting phenotypic plasticity and adaptation, as has been proposed in other polyploid systems [23,44]. IR events often occur in non-coding regions (relative to exon) or produce less drastic functional changes, allowing them to persist through evolution [32,45,46,47]. Subsequently, conserved sequence features associated with AS events were detected, which is important for regulating splicing efficiency, splice site recognition and the dynamic process of co-transcriptional splicing [48,49,50]. In all *Panax* species and the outgroup, the sequence features of skipped exons (lower GC content, shorter length) and retained introns (higher GC content, shorter length) were consistent with findings in model plants like wheat [40]. This conserved phenomenon suggests that these fundamental sequence constraints affecting splicing efficiency were established early in the evolution of the genus and have been maintained following WGD.

To analyze DAS between roots and leaves, we first compared PSI distributions in allotetraploid versus diploid species (Figure 3, Table 1). The results indicate that the process of polyploidization was accompanied by a widespread reshaping of splicing regulatory patterns. Furthermore, the divergence of PSI distribution among the allotetraploid species suggests that splicing regulation did not immediately stabilize after polyploidization but may have undergone continuous evolutionary adjustments. This post-polyploidization diversification pattern is similar to the process of regulatory network remodeling driven by genetic and epigenetic changes in other allopolyploids [34,51]. In plants, DAS is preferentially targeted to regulatory genes, including those encoding transcription factors, signaling kinases, and chromatin modifiers. This enrichment in the upstream regulatory network underscores a strategic evolutionary adaptation for achieving precise and rapid post-transcriptional control of gene expression [52,53,54]. GO enrichment analysis of DAS in the three allopolyploid *Panax* species (*P. ginseng*, *P. quinquefolius*, and *P. japonicus*) revealed that the most significantly enriched functions were concentrated in RNA binding, DNA metabolism, and stress signal response, suggesting that the evolution of AS in polyploid *Panax* follows a conserved and functionally biased trajectory. Despite significant lineage-specific divergence in the overall AS landscape, a series of conserved AS events were identified across diverse *Panax* species. Functional annotation of the conserved AS genes indicated that they were primarily involved in core regulatory functions, including chromatin remodeling [52], transcription factors (e.g., bHLH) [53], and RNA processing proteins (e.g., SR proteins) [54]. At the gene level, the *Panax* SCL33 (SR splicing factor) shows conserved intron retention in both leaves and roots across all five species (Tables S14 and S15). Given the central role of SR/SRPK pathways in splice-site selection, this conserved non-productive isoform likely mediates RNA-level gene dosage control, consistent with our observation that conserved AS events are enriched in RNA processing, which had been reported in *Arabidopsis* [10,26]. This finding suggests a role for AS in the precise modulation of central regulatory networks. Together with DAS between roots and leaves and species-specific gains in ES, A3 and A5, it suggests that *Panax* leverages AS to fine-tune regulatory nodes that coordinate stress and developmental programs, thereby linking conserved events in regulatory hubs to the environmental adaptability of the genus. This finding suggests a role for AS in the precise modulation of central regulatory networks. The ability to regulate the specific isoforms of these regulators via AS may influence global signaling and metabolic pathways [55,56]. In polyploid *Panax* species, this layer of post-transcriptional control is presumed to be critical for coordinating stress responses and developmental programs in response to genomic complexity.

In conclusion, the evolutionary history of *Panax* has been driven not only by whole-genome duplication but also by a significant diversification of its AS landscape. Allopolyploidization may have acted as a genomic catalyst, promoting the fusion of two distinct regulatory networks and providing a basis for genetic variation. While the core sequence rules governing splicing are conserved, the functional diversification of the transcriptome appears to be achieved through dynamic, species-specific changes in splicing events, particularly those involving genes at the top of regulatory hierarchies. This complex post-transcriptional regulatory layer likely constitutes one of the underlying mechanisms for the ecological adaptation and biochemical diversification observed in *Panax*.

## 4. Materials and Methods

### 4.1. Acquisition of RNA-Seq Datasets and Reference Genomes

This study utilized publicly available RNA sequencing (RNA-Seq) data. Raw paired-end RNA-Seq reads (FASTQ format) for *P. ginseng*, *P. quinquefolius*, *P. japonicus*, *P. stipuleanatus*, *P. notoginseng*, and the outgroup *D. carota* for leaf and root were downloaded from the China National Center for Bioinformation (CNCB), under BioProject accession numbers PRJNA900719 and PRJNA913450 [57,58]. The reference genomes were obtained from previously published and publicly available assemblies. The reference genomes for *P. ginseng*, *P. quinquefolius*, *P. japonicus*, and *P. stipuleanatus* were obtained from the China National Center for Bioinformation (CNCB) under BioProject accession PRJCA006678 [31]. The reference genome for *P. notoginseng* was acquired from the China National GeneBank DataBase (CNGBdb) under accession CNP0003588 [59]. The *D. carota* reference genome and corresponding annotations are available under the NCBI assembly accession GCF_001625215.2 (DH1 v.3) [60].

### 4.2. Phylogenetic Tree Construction

Genomes and corresponding annotations were acquired from NCBI and CNGB, then standardized through custom python (v3.8.19) pipelines implementing two critical filters: (i) retaining chromosome-level scaffolds, (ii) systematically converting chromosome identifiers to a “Chr” prefix format. Protein-coding sequences (CDS) were extracted by GffRead (v0.12.7) [61]. Subgenome assignments for *P. ginseng*, *P. japonicus*, and *P. quinquefolius* were leveraged to partition protein sequences into subgenomes A and B. All protein identifiers were systematically annotated with species and subgenome metadata to ensure traceability in downstream analyses. Orthogroup inference was performed with OrthoFinder (v2.5.5) [62] under the parameters: -t 64 -a 64 -A mafft -T fasttree -M msa -S blast -n out. The *D. carota* served as the outgroup in species phylogenetic tree, visualized with iTOL (v7, http://itol.embl.de/; accessed on 18 June 2025).

### 4.3. RNA-Seq Data Processing and Genome Alignment

Raw sequencing reads were processed by Trimmomatic (v0.39) [63] with default settings. Low-confidence bases (Phred < Q10) were discarded and adapters were clipped, yielding high-fidelity data sets for downstream analysis. Subsequently, reads were mapped to the corresponding reference genomes with the splice-aware aligner STAR (v2.7.11b) [64] in two-pass mode (--twopassMode Basic). The configuration permitted no more than two mismatches per read, and only uniquely aligned reads were retained. The SAMtools (v. 1.19.2) was used to retain the high mapping quality (Q20) results for downstream analyses [51].

### 4.4. Transcriptome Assembly and Annotation

Initial assembled transcripts for each biological replicate were constructed from alignment data using Scallop (v. 0.10.4) [65], with parameters set to --min_transcript_coverage 3 and --min_flank_length 5. To generate a comprehensive, species-specific annotation, the filtered assemblies from all biological replicates were consolidated by StringTie (v. 2.1.7) [66] (--merge -f 0.05 -T 1 -F 1 -m 200). Finally, GffCompare (v. 0.11.2) [61] was employed to compare the merged transcripts against the reference genome annotation and to extract splice pattern information. For all downstream analyses, only transcripts assigned the specific class codes (“=“, “j”, “c”, “k”, “m”, “n”) were retained.

### 4.5. Identification of Alternative Splicing Events

AS events were identified using SUPPA2 (v2.4) [7] with the annotation files generated through the aforementioned method. Four major types of AS events were included in the analysis: IR, ES, A5, and A3. To minimize false positives, the initial set of AS events was filtered using a custom Python (v3.8.19) script. Data from three biological replicates per sample were pooled, and only AS events supported by more than three reads containing the specific exon were retained, resulting in a final high-confidence set of AS events.

### 4.6. Analysis of GC Content and Length Features

GC content and length features were analyzed separately for exons involved in ES events and genomic background exons not associated with ES. Similarly, for IR events, features were compared between introns involved in IR and genomic background introns. The corresponding sequences were extracted from the reference genome using BEDTools (v2.31.1) [67]. GC content and length of exons and introns were then calculated using a custom Python (v3.8.19) script. Statistical comparisons were performed using the Wilcoxon test.

### 4.7. Differential Splicing Event Analysis

Alternative splicing (AS) was quantified by calculating the PSI for each splicing event. Events with 0 < PSI < 1 were counted to enumerate AS events present in leaf and root tissues across species. Differences in AS between leaves and roots within a species, as well as evolutionary dynamics across species of different ploidy levels, were assessed using ΔPSI (|PSI_2_−PSI_1_|). For pairwise comparisons, a binomial test was applied based on the numbers of reads supporting exon inclusion versus exon skipping. Significant differential splicing events (DSEs) were defined by the thresholds |∆PSI| > 0.2 and *P* < 0.05. Differential splicing events (DSEs) were classified into three categories. A “gain” AS event (gain ASE) was identified when PSI_leaf_ > 0.95 or <0.05, while PSI_root_ was between 0.05 and 0.95. Conversely, a “loss” AS event (lost ASE) was recognized when PSI_root_ > 0.95 or < 0.05, while PSI_leaf_ was between 0.05 and 0.95. The remaining DSEs were categorized as differential alternative splicing events.

### 4.8. Conservation Analysis of Alternative Splicing

The identification of homologous exons among orthologous gene pairs was initiated by extracting all exon DNA sequences from single-copy orthogroups derived from OrthoFinder (v2.5.5) [62]. Each sequence was annotated with a “gene and exon number” identifier. Reciprocal BLASTN (v2.5.0) alignments were conducted between exons from the same orthologous gene family across any two species to detect reciprocal best BLAST hits (RBH). The resulting alignments were filtered using a custom Python (v3.8.19) script to retain only exon pairs satisfying an e-value threshold of ≤ 1 × 10^−5^ and a minimum alignment coverage of 60% for both sequences, thereby establishing a cross-species exon homology reference set for subsequent conservation analysis (Figure 5).

Conservation of alternative splicing events was assessed by mapping species-specific AS events to the exon homology reference set. For two orthologous genes, denoted ‘a’ from species A and ‘b’ from species B, an ES event was considered conserved if the following criteria were met: (i) both ES events occurred within the same orthologous gene family; (ii) the skipped exons constituted an RBH pair between the two species; and (iii) the flanking upstream and downstream exons involved in the ES event also formed RBH pairs with their counterparts in the orthologous gene. For terminal exons, the RBH requirement was applied only to the single available flanking exon. For IR events, conservation was evaluated by comparing detected IR events against the exon homology reference set. An IR event was classified as conserved if both species exhibited retention of the intron located between the same pair of flanking exons (e.g., exon1–intron–exon2), and each flanking exon formed an RBH with its orthologous counterpart. An IR event was considered non-conserved if IR was detected in only one species, with the other species showing a spliced junction between the two exons or lacking evidence for the event. Events were also deemed non-conserved if IR was observed in both species but the flanking exons could not be reliably matched as RBHs. Candidate conserved AS events were merged and organized across species using a custom python (v3.8.19) script based on orthologous gene family and exon number identifiers. Final statistical summaries and visualizations were generated using custom Python (v3.8.19) and R scripts to characterize the cross-species distribution and patterns of conserved splicing events.

## Figures and Tables

**Table 1:** Pairwise comparisons of PSI distributions by Kolmogorov–Smirnov test for AS events in leaf and root tissues among *Panax* species.

Pairwise Comparison	Leaf (*p*-Value)	Root (*p*-Value)
*P. ginseng* vs. *P. quinquefolius*	8.90 × 10^−4^	5.78 × 10^−1^
*P. ginseng* vs. *P. japonicus*	2.17 × 10^−190^	4.44 × 10^−19^
*P. quinquefolius* vs. *P. japonicus*	1.23 × 10^−147^	7.69 × 10^−16^
*P. notoginseng* vs. *P. stipuleanatus*	2.12 × 1^−1^	1.20 × 10^−1^
*P. ginseng* vs. *P. notoginseng*	6.57 × 10^−10^	5.83 × 10^−3^
*P. quinquefolius* vs. *P. notoginseng*	6.13 × 10^−4^	1.88 × 10^−2^
*P. japonicus* vs. *P. notoginseng*	5.17 × 10^−85^	3.05 × 10^−19^
*P. ginseng* vs. *P. stipuleanatus*	5.49 × 10^−7^	1.64 × 10^−3^
*P. quinquefolius* vs. *P. stipuleanatus*	1.06 × 10^−1^	1.66 × 10^−2^
*P. japonicus* vs. *P. stipuleanatus*	4.36 × 10^−105^	7.29 × 10^−20^

## Data Availability

Data are available in the article’s Supporting Information.

## Supplementary Materials

The following supplemental information can be downloaded at https://www.mdpi.com/article/10.3390/plants14213301/s1, Figure S1. Maximum likelihood phylogenetic tree depicting evolutionary relationships among five *Panax* species with *D. carota* as the outgroup; Figure S2. Three-cluster conservation of AS events on orthologous genes across *Panax* species; Figure S3. Four-cluster conservation of AS events on orthologous genes across *Panax* species; Figure S4. Two-cluster conservation of AS events on orthologous genes across subgenomes of *Panax* species; Figure S5. Three-cluster conservation of AS events on orthologous genes across subgenomes of *Panax* species; Table S1. The number of AS events in the leaf of six species; Table S2. The number of AS events in the root of six species; Table S3. The gene number of AS events in the leaf of six species; Table S4. The gene number of AS events in the root of six species; Table S5. Number of ASEs (Alternative Splicing Event) of each ASG (Alternative Splicing Gene) in the leaf of six species; Table S6. Number of ASEs (Alternative Splicing Event) of each ASG (Alternative Splicing Gene) in the root of six species; Table S7. Quantitative–qualitative composition of differentially spliced events (DSEs) of *P. ginseng*; Table S8. Quantitative–qualitative composition of differentially spliced events (DSEs) of *P. japonicus*; Table S9. Quantitative–qualitative composition of differentially spliced events (DSEs) of *P. quinquefolius*; Table S10. Quantitative–qualitative composition of differentially spliced events (DSEs) of *P. notoginseng*; Table S11. Quantitative–qualitative composition of differentially spliced events (DSEs) of *P. stipuleanatus*; Table S12. Conserved AS of leaf between five *Panax* species; Table S13. Conserved AS of root between five *Panax* species; Table S14. The gene of conserved AS in leaf between five *Panax* species; Table S15. The gene of conserved AS in root between five *Panax* species.
