# Supplementary material for: Conservation and Remodeling of Alternative Splicing Landscapes in the Evolution of Panax
Source: Plants (Basel). 2025 Oct 29;14(21):3301. doi: 10.3390/plants14213301 (PMC12609446; doi:10.3390/plants14213301)
Supplement: Supplementary file 1 [file plants-14-03301-s001.zip › Supporting Information.pdf]

Supporting Information

Article title: Conservation and Remodeling of Alternative Splicing Landscapes in the Evolution of Panax

Jing Zhao[1,4†], Xiangru Meng[1,4†],Peng Di[1,4], Junbo Rong[1,4], Hongwei Xun[6], Siwen Zheng[1,4], Juzuo Li[5*], Jian Zhang[1,2,3*], Ying-Ping Wang[1,4*]

The following Supporting Information is available for this article:

Figure S1. Maximum likelihood phylogenetic tree depicting evolutionary relationships among five *Panax* species with *D. carota* as the outgroup.

Figure S2. Three-cluster conservation of AS events on orthologous genes across *Panax* species.

Figure S3. Four-cluster conservation of AS events on orthologous genes across *Panax* species.

Figure S4. Two-cluster conservation of AS events on orthologous genes across subgenomes of *Panax* species.

Figure S5. Three-cluster conservation of AS events on orthologous genes across subgenomes of *Panax* species.

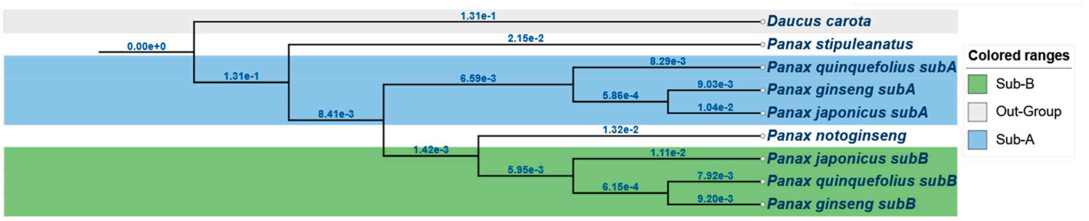

Figure S1. Maximum likelihood phylogenetic tree depicting evolutionary relationships among five *Panax* species with *D. carota* as the outgroup. Homeologous subgenomes are resolved into two distinct clades, designated Subgenome A (blue) and Subgenome B (green).

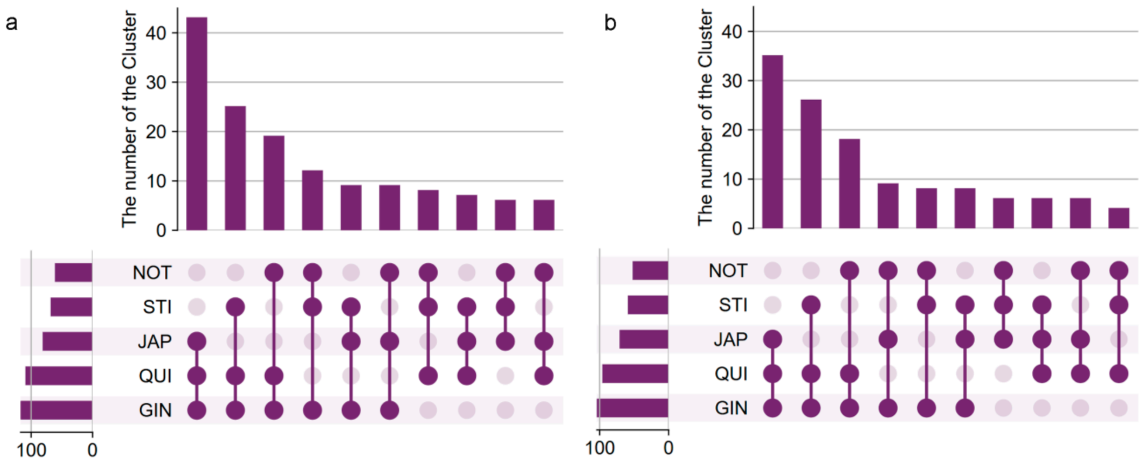

Figure S2. Three-cluster conservation of AS events on orthologous genes across *Panax* species. (a) Conservation clusters of IR events in leaf tissue. (b) Conservation clusters of IR events in root tissue. The upper bar chart indicates the number of AS event clusters corresponding to the intersection sets connected below; the lower section, represented by solid circles and connecting lines, denotes the specific *Panax* species; the horizontal bars on the left represent the total number of AS event clusters contained within each individual set. Species abbreviations: GIN: ***P. ginseng***; QUI: ***P. quinquefolius***; JAP: ***P. japonicus***; NOT: ***P. notoginseng***; STI: ***P. stipuleanatus***.

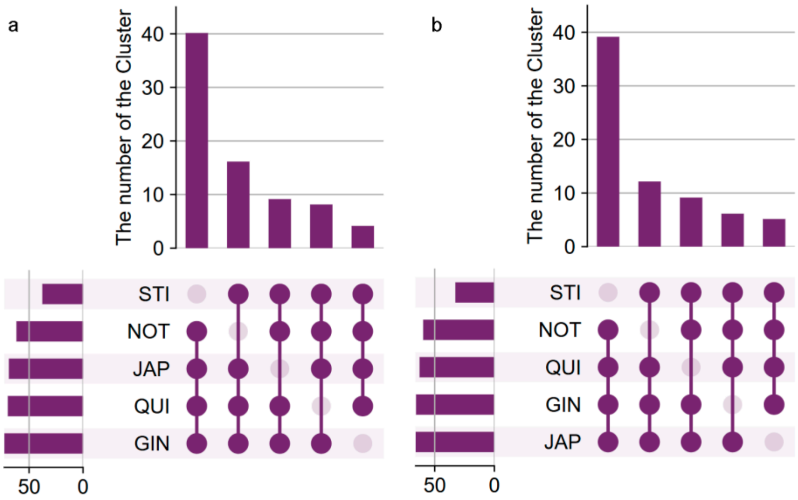

Figure S3. Four-cluster conservation of AS events on orthologous genes across *Panax* species. (a) Conservation clusters of IR events in leaf tissue. (b) Conservation clusters of IR events in root tissue. The upper bar chart indicates the number of AS event clusters corresponding to the intersection sets connected below; the lower section, represented by solid circles and connecting lines, denotes the specific *Panax* species; the horizontal bars on the left represent the total number of AS event clusters contained within each individual set. Species abbreviations: GIN: ***P. ginseng***; QUI: ***P. quinquefolius***; JAP: ***P. japonicus***; NOT: ***P. notoginseng***; STI: ***P. stipuleanatus***.

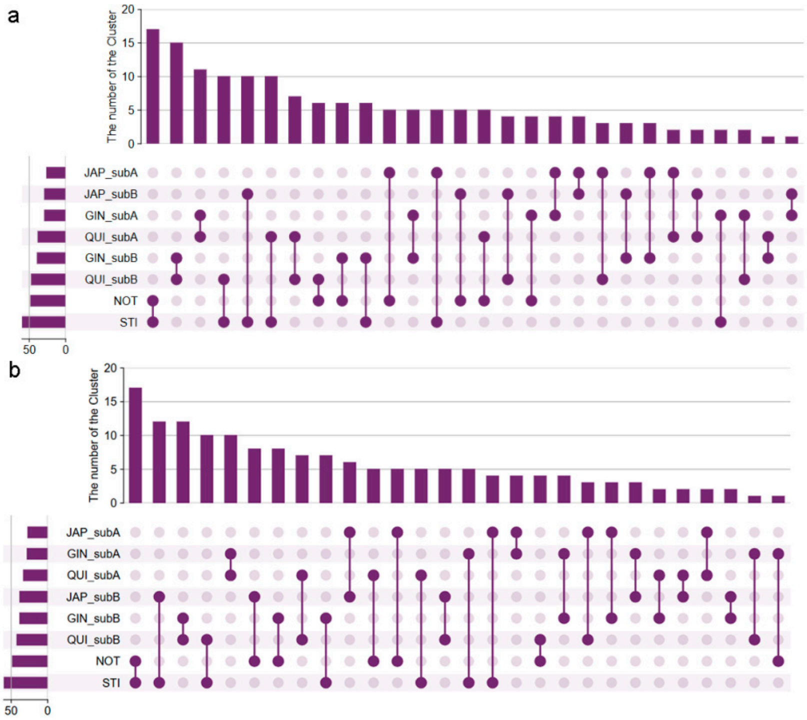

Figure S4. Two-cluster conservation of AS events on orthologous genes across subgenomes of *Panax* species. (a) Conservation clusters of IR events in leaf tissue. (b) Conservation clusters of IR events in root tissue. The upper bar chart indicates the number of AS event clusters corresponding to the intersection sets connected below; the lower part, represented by solid circles and connecting lines, denotes the specific combinations of subgenomes; the horizontal bars on the left represent the total number of AS event clusters contained within each individual set. Species abbreviations: GIN: ***P. ginseng***; QUI: ***P. quinquefolius***; JAP: ***P. japonicus***; NOT: ***P. notoginseng***; STI: ***P. stipuleanatus***. SubA and SubB indicate the two subgenomes of each species.

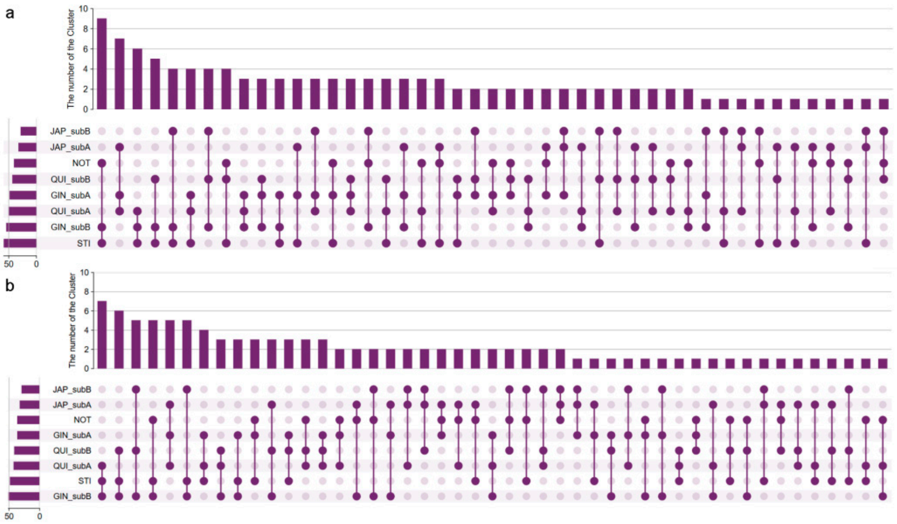

Figure S5. Three-cluster conservation of AS events on orthologous genes across subgenomes of *Panax* species. (a) Conservation clusters of IR events in leaf tissue. (b) Conservation clusters of IR events in root tissue. The upper bar chart indicates the number of AS event clusters corresponding to the intersection sets connected below; the lower part, represented by solid circles and connecting lines, denotes the specific combinations of subgenomes; the horizontal bars on the left represent the total number of AS event clusters contained within each individual set. Species abbreviations: GIN: ***P. ginseng***; QUI: ***P. quinquefolius***; JAP: ***P. japonicus***; NOT: ***P. notoginseng***; STI: ***P. stipuleanatus***. SubA and SubB indicate the two subgenomes of each species.
